# APOBEC3 signature mutations in chronic lymphocytic leukemia

**DOI:** 10.1038/leu.2014.160

**Published:** 2014-06-10

**Authors:** S Rebhandl, M Huemer, F J Gassner, N Zaborsky, D Hebenstreit, K Catakovic, E M Grössinger, R Greil, R Geisberger

**Affiliations:** 1 Department of Internal Medicine III with Hematology, Medical Oncology, Hemostaseology, Infectious Diseases, Rheumatology, Oncologic Center, Laboratory for Immunological and Molecular Cancer Research, Paracelsus Medical University, Salzburg, Austria; 2 School of Life Sciences, University of Warwick, Coventry, UK

Cytidine deaminases of the APOBEC family (ApoB mRNA editing catalytic subunit) generate targeted damage in nucleic acids by deaminating cytosins to uracils. The catalytically active family members are APOBEC1, APOBEC3 proteins (which comprise APOBEC3A, APOBEC3B, APOBEC3C, APOBEC3D, APOBEC3F, APOBEC3G, APOBEC3H) and activation-induced deaminase (AID) (reviewed in Conticello^1, 2^). APOBEC1 deaminates the mRNA for Apolipoprotein B thereby generating ApoB48, which mediates the absorption of dietary lipid from the intestine.^3^ Whereas APOBEC3 proteins are implicated in the natural defense against retroviruses and mobile elements by mutating and thereby inactivating retroviral single stranded DNA intermediates, AID mediates somatic hypermutation and class switch recombination of immunoglobulin genes in B cells.^4^ As it was shown that AID can cause substantial off-target DNA damage, AID has been implicated in B-cell lymphomagenesis by mutating oncogenes and by mediating chromosomal translocations as side product of aberrant class switch recombination.^5^ In chronic lymphocytic leukemia (CLL), previous reports have described the presence of AID transcripts in a subset of CLL patients where AID-expressing CLL patients exhibited a significantly decreased time to treatment and overall survival.^6^ Paradoxically, AID levels were higher in CLL cells expressing unmutated immunoglobulin variable domains (IgV-UM) encoding the B-cell antigen receptor, which predicts for poor prognosis, whereas CLL cells with somatically hypermutated IgV (IgV-Mut) tend to have lower AID transcript abundances.^7^ Also, AID expression associates with complex genomic aberrations and with Richter’s transformation to a more aggressive disease,^8^ providing strong evidence that AID is implicated in clonal evolution and pathogenesis in CLL. However, recent studies have revealed that other APOBEC family members are as well implicated in cancer development by inducing genome wide C>U deaminations that turn to C>T transition mutations after DNA replication.^9^ It was found that APOBEC-mediated mutations can lead to mutation showers (termed kataegis) in which multiple mutations are spaced by only one to several hundred nucleotides.^10^

In CLL, kataegic events were recently described that exhibit a signature of AID-mediated hypermutation.^11^ These clustered mutations are mostly but not exclusively found within the Ig locus and are restricted to IgV-Mut CLL cases.^11^ To compare clustered mutations inside and outside the Ig locus, we reanalyzed CLL whole genome sequencing data from Puente and coworkers, who sequenced two IgV-UM and two IgV-Mut CLL samples (Supplementary Table 6 from Puente *et al.*^12^). We thereby found that genome-wide mutations had a mean intermutational distance from 2.8 Mb (95% confidence interval: 2.698–2.937 Mb), which was similar for IgV-Mut and IgV-UM samples (Figure 1a). By searching for clustered mutations with intermutational distances below 10 kb, we noticed that while the two IgV-UM samples did not harbor mutation foci (defined as ⩾3 mutations each spaced by ⩽10 kb), the two IgV-Mut samples exhibited 19 clusters with a total of 113 (5.4% of total) mutations where ⩾3 mutations were spaced with an intermutational distance below 10 kb (Figure 1a). From these clusters, 49 mutations (43%) were located within the Ig locus and 64 (57%) were located outside the Ig locus (Figure 1a and Supplementary Table 1). In contrast to kataegis described in breast cancer,^11^ the clustered mutations found in CLL do not have a particular bias toward C>T or C>G mutations, neither at Ig nor at non-Ig clusters Supplementary Table 1), but rather have a mutation feature typical for somatic hypermutation where AID-mediated cytidine deamination is processed by error prone polymerases.^4, 11^ In line with this, C>T transitions within clustered mutations at the Ig loci were confined to the AID hot spot motif WRCY (W=A or T; R=A or G; Y=C or T; Figure 1c). In contrast, C>T transitions at mutation clusters outside the Ig locus were primarily found at TCW motifs, which is no AID but an APOBEC3 mutation motif (Figure 1c).^10^ Genome-wide unclustered C>T mutations outside mutation foci did not show a certain motif bias, neither when all CpGs or CpG islands were excluded from the analysis, as methylated cytosines (which are confined to CpG dinucleotides) are more prone to spontaneous deamination than normal cytosines^13^ (Figure 1b). Apart from the APOBEC3 motif at C>T transitions within clustered mutations at non-Ig loci, the spectrum of mutations was quite similar to hypermutation at Ig loci, with no apparent sequence bias (Supplementary Table 1). Thus, it is tempting to speculate that in CLL, APOBEC3 might operate in combination with factors of the hypermutation machinery to induce off-target DNA damage at non-Ig loci.

As our mutation analysis revealed that C>T transitions within small mutation clusters occur at APOBEC3 recognition motifs outside the Ig loci in CLL, we next asked whether any APOBEC members are expressed in CLL samples. We therefore measured transcript levels of catalytically active members APOBEC3A to APOBEC3H using SYBR green quantitative reverse transcription (qRT)-PCR in a set of 10 IgV-Mut and 8 IgV-UM CLL samples and compared values with that from purified B cells of 5 healthy controls. As shown in Figure 2a, we found that APOBEC3A, APOBEC3B and APOBEC3H were expressed in CLL samples with a slight upregulation compared with healthy controls (Figure 2a). In addition, we observed a slight but significant difference in APOBEC3A, APOBEC3B and APOBEC3H expression values between IgV-Mut or IgV-UM samples (APOBEC3A: median 2.87 vs 1.92, *P*=0.016; APOBEC3B: median 1.37 vs 0.82, *P*=0.034; APOBEC3H: median 4.66 vs 2.77, *P*=0.021; Figure 2a). We subsequently tested protein expression of APOBEC3A, APOBEC3B and APOBEC3H by immunoblotting and found bands corresponding to APOBEC3A and APOBEC3B in almost any CLL sample irrespective of IgV mutation status (Figure 2b). Although median expression values of APOBEC3A and APOBEC3B were 1.5 and 1.7 times higher in IgV-Mut compared with IgV-UM CLL samples in qRT-PCR, this difference was too small to be discerned in immunoblotting on the level of protein. APOBEC3H, albeit showing highest expression in qRT-PCR, did not show detectable protein amounts in immunoblotting. A faint band for APOBEC3H that appeared in MEC1 cell lysates served as positive control (Figure 2b). Upon stimulation of CLL cells with CpG, APOBEC3B protein was upregulated, while APOBEC3A remained quite constant or even showed reduced band intensities in immunoblotting (Figure 2b). The APOBEC member AID was only detectable on *in vitro* stimulation of cells with CpG before cell lysis in one IgV-Mut and one IgV-UM sample, respectively (Figure 2b).

Overall, our data show that aside of AID, also APOBEC3 deaminases are likely implicated in mediating off-target mutations in CLL. We show that APOBEC3A and APOBEC3B are more abundant on protein level in CLL than in AID, and clustered genomic C>T mutations outside the Ig locus occur at APOBEC3 hot spot motifs in IgV-Mut CLL. As these clustered mutations were confined to IgV-Mut samples, we initially suspected that APOBEC3 members were differentially expressed according to the IgV mutation status. However, although APOBEC3A, APOBEC3B and APOBEC3H transcripts were significantly lower expressed in IgV-UM samples, it is questionable whether this small difference has biological significance, as APOBEC3A and APOBEC3B were easily detectable on the protein level in both IgV-Mut as well as IgV-UM samples. However, as it was shown that APOBEC3 levels correlate with APOBEC3-induced mutations,^14^ it is conceivable that even small differences in APOBEC3 expression levels could account for the observed difference in APOBEC3-induced clustered mutations in IgV-Mut vs IgV-UM CLL (Figure 2a). Alternatively, as recent studies have revealed that apart from APOBEC expression additional factors of the DNA repair machinery^10^ are required for inducing kataegis, our data could suggest that any of these factors are not expressed in IgV-UM samples, similar to the observation that AID expression in IgV-UM CLL samples is not sufficient to diversify IgV genes.^7^ The absence of any of these factors could either alter the targeting of APOBEC3 to the DNA or otherwise affect the error prone DNA repair of APOBEC3-induced DNA lesions. Hence, it is conceivable that APOBEC3 might operate non-processively in IgV-UM CLL, resulting in the generation of unclustered mutations as opposed to clustered kataegic events in IgV-Mut CLL. From our data we conclude that aside of AID, also APOBEC3 should be considered as a source for genomic mutations in CLL. As CLL has a substantial intraclonal heterogeneity,^15^ we suspect that ongoing APOBEC3 expression might contribute to this genetic complexity by continuously increasing the mutation load, thereby accelerating clonal evolution to a more aggressive or chemorefractory disease. We further propose that analyzing IgV-Mut vs IgV-UM CLL will likely yield a deeper insight into mechanisms that underlie the initiation of APOBEC3-mediated clustered vs unclustered DNA mutations in cancer in general.

## Figures and Tables

**Figure 1:**
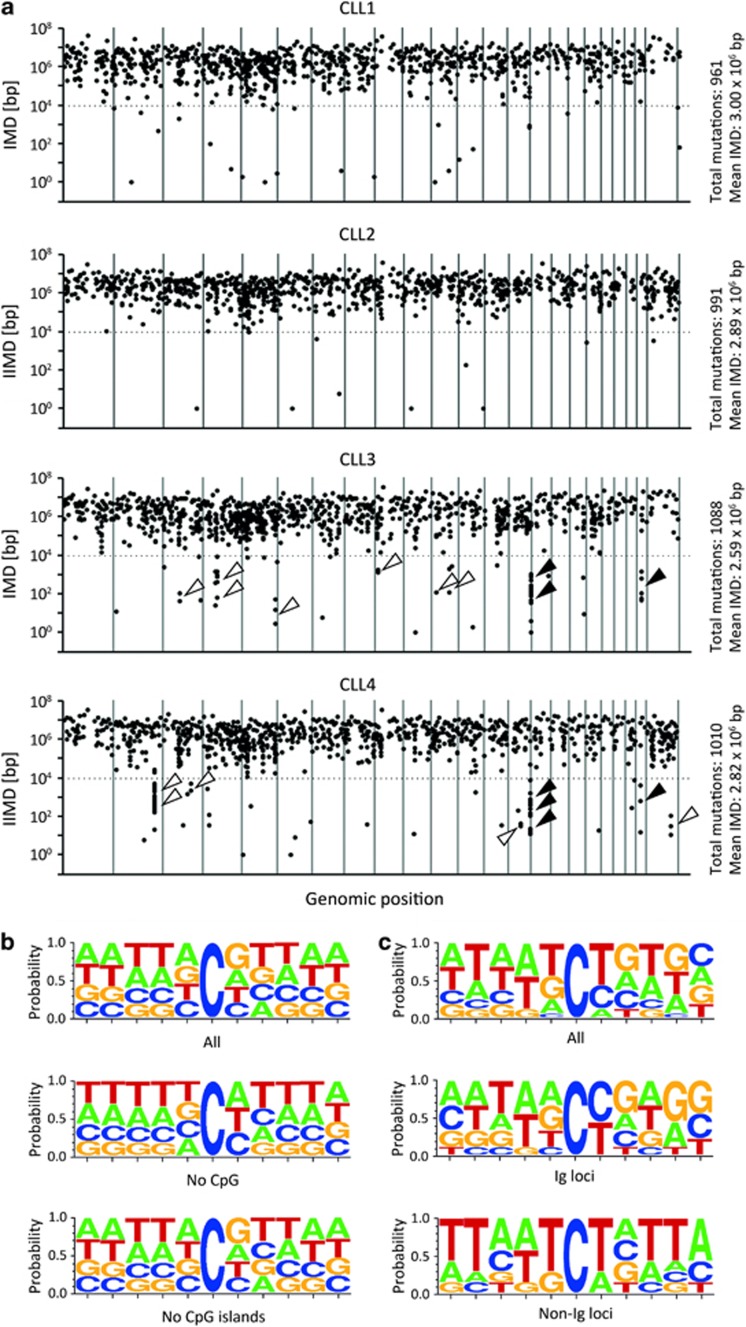
Clustered mutations are present in IgV-Mut CLL samples. (**a**) Analysis of whole genome sequencing data by rainfall plots shows intermutational distances (IMDs) in four individual CLL cases with unmutated (CLL1 and 2) and mutated IgV (CLL3 and 4). Mutations are shown as dots with the *y* axis giving the distance to the next downstream mutation on the same chromosome. The genomic position of the respective mutation is given on the *x* axis, with chromosomes (1–22, *x*,*y*) spaced by vertical lines. Total mutations and mean IMDs are indicated to the right of each rainfall plot. Clustered mutations (defined as ⩾3 mutations spaced by ⩽10kb) are indicated with arrows. Clustered mutations inside the Ig loci are marked with black arrows and outside Ig loci with white arrows. Local sequence context of (**b**) unclustered and (**c**) clustered C>T transitions from CLL data from (**a**). (**b**) The probability of occurrence of individual bases upstream and downstream of a C mutated to T is shown for all mutated Cs (upper panel), for Cs outside CpGs (middle panel) and outside CpG islands (lower panel) as defined by the UCSC Table Browser tool. (**c**) Sequence context of all clustered C>T mutations (total) are shown and for clustered C>T mutations at Ig (middle panel) and non-Ig loci (lower panel).

**Figure 2:**
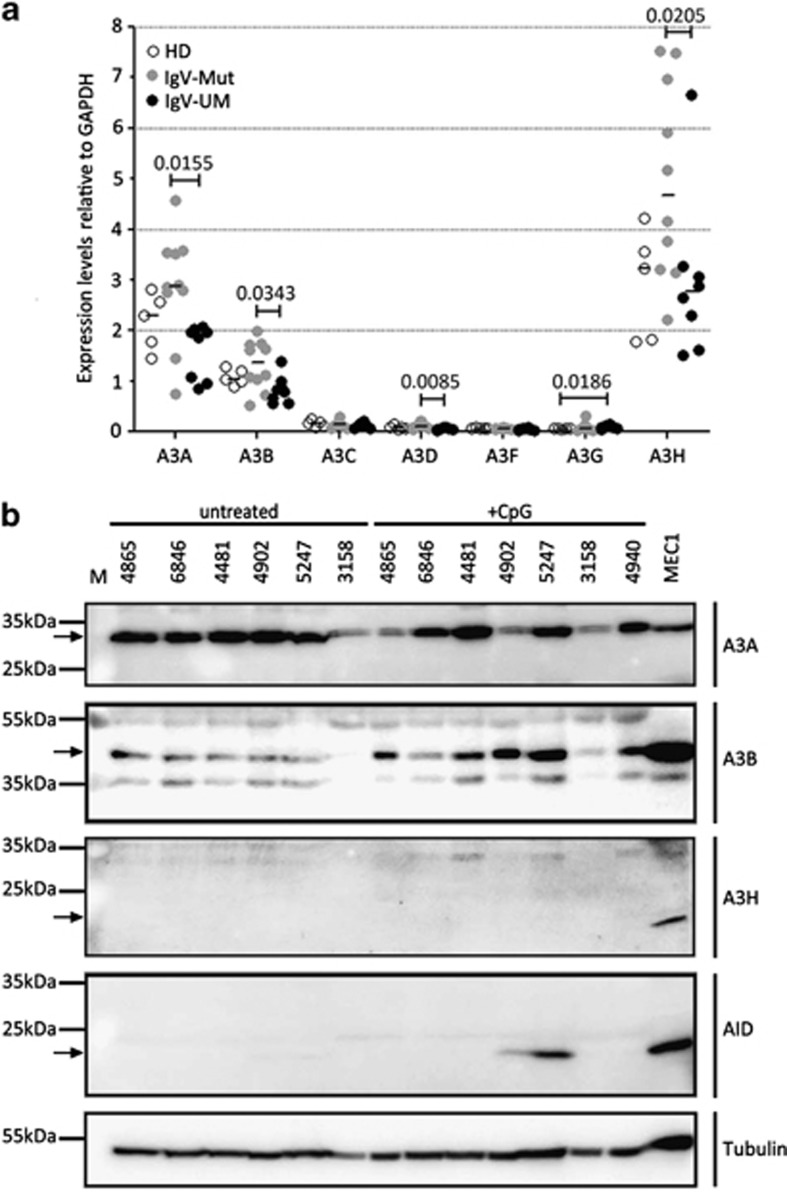
APOBEC3 family members are expressed in CLL. (**a**) SYBR green qRT-PCR data from cDNA of 10 IgV-Mut, 8 IgV-UM CLL samples and 5 healthy controls (HD) using APOBEC3A-H (abbreviated: A3A-H)-specific primer sets normalized to glyceraldehyde 3-phosphate dehydrogenase (GAPDH) levels (scatter dot blot with median values indicated as bars). Statistically significant values are indicated within the graph. (**b**) Analysis of APOBEC3A, APOBEC3B and APOBEC3H expression levels in peripheral blood mononucleated cells from primary IgV-Mut (IDs 4865, 6846, 4902, 3185, 4940) and IgV-UM (IDs 4481, 5247) patients were determined by immunoblotting after 8 days under cell culture conditions with (+CpG) or without (untreated) CpG treatment. Arrows to the left indicate specific bands. Tubulin was used as a loading control.

## Supplementary Material

Supplementary Table1

Supplementary Methods

Supplementary Patient List
