# Supplementary material for: APOBEC3 signature mutations in chronic lymphocytic leukemia
Source: Leukemia. 2014 Jun 10;28(9):1929–32. doi: 10.1038/leu.2014.160 (PMC4140768; doi:10.1038/leu.2014.160)
Supplement: Supplementary Table1 [file leu2014160x1.doc]

**Suppl Table 1.** Mutational pattern of clustered mutations in CLL.

|  | clustered mutations | | | | | |
| --- | --- | --- | --- | --- | --- | --- |
|  | total | | at Ig loci | | at Non-Ig loci | |
| Mutation | No. of events | % of total | No. of events | % of total | No. of events | % of total |
| A→G/T→C | 36 | 31.9% | 15 | 30.6% | 21 | 32.8% |
| A→C/T→G | 17 | 15.0% | 5 | 10.2% | 12 | 18.8% |
| A→T/T→A | 24 | 21.2% | 6 | 12.2% | 18 | 28.1% |
| G→C/C→G | 11 | 9.7% | 10 | 20.4% | 1 | 1.6% |
| G→T/C→A | 9 | 8.0% | 4 | 8.2% | 5 | 7.8% |
| C→T/G→A | 16 | 14.2% | 9 | 18.4% | 7 | 10.9% |
| Total | 113 | 100% | 49 | 100% | 64 | 100% |

clustered mutations (≥3 each spaced by less than 10kb) from Puente et al were separately analyzed depending on whether there are positioned within or outside Ig loci.
