# Supplementary material for: APOBEC3 signature mutations in chronic lymphocytic leukemia
Source: Leukemia. 2014 Jun 10;28(9):1929–32. doi: 10.1038/leu.2014.160 (PMC4140768; doi:10.1038/leu.2014.160)
Supplement: Supplementary Methods [file leu2014160x2.doc]

**Supplementary Information**

**Methods**

**Human CLL samples**

Peripheral blood samples from CLL patients from our outpatient care facility at the III^rd^ Medical Department were collected upon informed consent in accordance with the Declaration of Helsinki and upon approval by the Ethics Committee of Salzburg, Austria (Ref. No. 415-E/1287/8-2011). Peripheral blood mononuclear cells (PBMCs) were separated by density centrifugation using Biocoll (Biochrom AG). The immunoglobulin heavy chain variable region (IgVH) mutational status, CD38, Zap-70 and VLA-4 expression were determined as previously described (1). For detection of genomic aberrations, interphase fluorescent in situ hybridization (FISH) analysis using the multicolor probe set (Abbott/Vysis) was performed. CLL cells were isolated from PBMCs using magnetic beads and purity was assessed by CD5CD19 staining using flow cytometry (purity for samples used for qRT-PCR was ≥95%). B cells from healthy controls were isolated from PBMCs using anti-CD19 magnetic beads. (a detailed patient list is shown as SI Table 2).

**SYBR green qRT-PCR**

RNA was isolated from purified CLL samples (Qiagen) and 1^st^ strand cDNA was generated (iScript, Bio-Rad). For SYBR green RT-PCR the LightCycler® FastStart DNA Master SYBR Green I kit (Roche) was used. Expression values of APOBECs were calculated as fold change compared to GAPDH (2^-dCt). Primers used for SYBR-green qRT-PCR were used according to (2): APOBEC3A (RSH2742 gagaagggacaagcacatgg RSH2743 tggatccatcaagtgtctgg), APOBEC3B (RSH3220 gaccctttggtccttcgac RSH3221 gcacagccccaggagaag), APOBEC3C (RSH3085 agcgcttcagaaaagagtgg RSH3086 aagtttcgttccgatcgttg), APOBEC3D (acccaaacgtcagtcgaatc RSH2750 cacatttctgcgtggttctc), APOBEC3F (RSH2751 ccgtttggacgcaaagat RSH2752 ccaggtgatctggaaacactt), APOBEC3G (RSH2753 ccgaggacccgaaggttac RSH2754 tccaacagtgctgaaattcg), APOBEC3H (RSH2757 agctgtggccagaagcac RSH2758 cggaatgtttcggctgtt). For GAPDH primers RG518 (gaaggtgaaggtcggagtc) and RG519 (gaagatggtgatgggatttc) were used.

**Immunoblotting**

For immunoblotting, PBMCs from primary CLL samples were either left untreated or cultured for 8 days in RPMI1640 supplemented with 10% fetal calf serum, 50µM ß-mercaptoethanol, 100µg/mL streptomycin and 100U/ml penicillin. For in vitro activation cells were stimulated with 1µM CpG phosphorothioate oligonucleotide DSP30 (3) (MWG Eurofins). For whole cell lysates cells were directly lysed in reducing sample buffer (62.5mM Tris-HCl (pH 7.5), 10% glycerol, 5% ß-mercaptoethanol, 2% SDS and 0.005% bromphenol blue), heat denaturated at 95°C for 5min and loaded onto 12% polyacrylamide gels. Proteins were transferred onto PVDF-membranes and detected using specific antibodies for APOBEC3B, APOBEC3B and APOBEC3H (pAb, Novus Biologicals), AID (mAb EK2 5G9, Cell Signaling), and Tubulin (mAb B-5-1-2, Sigma), followed by secondary HRP-conjugated antibodies (pAb anti rat HRP #7097, Abcam; pAb anti mouse HRP, DAKO, pAb anti rabbit HRP #7074, Cell Signaling).

**Bioinformatics**

For analysis of genome wide C>T transitions, Supplementary Table 6 of (4) containing the positions of the mutations and neighboring sequences for all four samples (‘CLL1’ to ‘4’) was downloaded. C>Y and G>R mutations and five neighboring bases on each side of the mutations were extracted. The G>R set was converted to the reverse complement. The nucleotide compositions of the resulting combined 1226 sequences were then analysed with the Weblogo online tool (5). The analysis was repeated with sequences that were depleted of those containing a ‘G’ nucleotide after the central mutation (resulting in 785 sequences). In addition, the original table was converted to a BED format file containing the positions of the mutations with ten neighboring bases on each side. This file was uploaded to the UCSC genome browser (http://genome-euro.ucsc.edu) to remove all sequence stretches that had any overlap with CpG islands using the Table Browser tool (resulting in 1208 sequences). The remaining sequences were analysed for their nucleotide composition in the same way as described above. Custom Perl scripts were used at all steps.

**Statistics**

Intermutational distances (IMD) were calculated from Supplementary Table 6 from Puente et al (4) using Excel. For rainfall plots, IMDs were plotted against position of mutation using graph pad. Statistical analysis was performed with GraphPad Prism 5. Mann-Whitney test (non-normally distributed samples) was used to determine significance.

Reference List

(1) Asslaber D, Pinon JD, Seyfried I, Desch P, Stocher M, Tinhofer I, et al. microRNA-34a expression correlates with MDM2 SNP309 polymorphism and treatment-free survival in chronic lymphocytic leukemia. Blood 2010 May 27;115(21):4191-7.

(2) Burns MB, Lackey L, Carpenter MA, Rathore A, Land AM, Leonard B, et al. APOBEC3B is an enzymatic source of mutation in breast cancer. Nature 2013 Feb 21;494(7437):366-70.

(3) Liang H, Nishioka Y, Reich CF, Pisetsky DS, Lipsky PE. Activation of human B cells by phosphorothioate oligodeoxynucleotides. Journal of Clinical Investigation 1996 Sep 1;98(5):1119-29.

(4) Puente XS, Pinyol M, Quesada V, Conde L, Ordonez GR, Villamor N, et al. Whole-genome sequencing identifies recurrent mutations in chronic lymphocytic leukaemia. Nature 2011 Jul 7;475(7354):101-5.

(5) Crooks GE, Hon G, Chandonia JM, Brenner SE. WebLogo: A sequence logo generator. Genome Research 2004 Jun;14(6):1188-90.
