# Supplementary material for: APOBEC3 signature mutations in chronic lymphocytic leukemia
Source: Leukemia. 2014 Jun 10;28(9):1929–32. doi: 10.1038/leu.2014.160 (PMC4140768; doi:10.1038/leu.2014.160)
Supplement: Supplementary Patient List [file leu2014160x3.pdf]

ID	Age at diagnosis [yrs]	Age at sampling [yrs]	Treatment status at sampling date	Sex	Chromosomal aberrations	IgVH Status	ZAP-70	VLA-4	CD38	RAI
3158	73	82	treated	female	normal	mutated	low	n.d.	high	0-2
4481	64	67	chemonaive	male	13q	unmutated	n.d.	n.d.	low	0
4865	63	69	chemonaive	male	13q	mutated	low	low	low	2
4902	69	79	treated	male	13q	mutated	low	low	low	0
4940	37	55	treated	male	13q	mutated	low	low	low	2
5247	80	81	chemonaive	female	n.d.	unmutated	n.d.	high	high	0
6846	76	86	chemonaive	male	13q	mutated	low	low	low	0
7073	59	64	treated	female	13q	mutated	low	low	low	1
7075	58	62	chemonaive	male	normal	mutated	low	low	low	0
7097	72	77	treated	male	tri12	unmutated	high	high	low	1
7116	75	79	chemonaive	female	normal	unmutated	n.d.	low	high	0-1
7152	49	57	treated	male	normal	unmutated	high	high	high	2+3
7177	62	69	treated	male	13q17p, tri12	unmutated	high	high	high	2-4
7461	64	66	chemonaive	female	tri12	unmutated	low	high	low	1
7500	70	75	treated	female	13q	unmutated	high	high	high	0
7593	78	81	chemonaive	male	13q	mutated	low	low	low	1
7775	59	83	treated	male	normal	unmutated	n.d.	high	high	2
7805	65	70	treated	male	11q13q	unmutated	low	low	high	1
7822	76	84	chemonaive	male	13q17p	mutated	low	low	low	0
7847	53	65	chemonaive	male	13q	mutated	high	low	low	1
7848	78	79	chemonaive	female	13q	mutated	n.d.	low	low	1
7893	46	49	chemonaive	male	normal	mutated	low	low	low	0
7906	61	75	treated	male	13q	mutated	low	high	low	2-4
7997	70	85	chemonaive	female	tri12	mutated	low	high	low	0-1
8039	57	63	chemonaive	male	normal	mutated	low	high	low	1

n.d.: not determined
yrs: years

Patient list
